# The developing brain: Challenges and opportunities to promote school readiness in young children at risk of neurodevelopmental disorders in low- and middle-income countries

**DOI:** 10.3389/fped.2022.989518

**Published:** 2022-10-21

**Authors:** Mijna Hadders-Algra

**Affiliations:** University of Groningen, University Medical Center Groningen, Department of Pediatrics, Division of Developmental Neurology and University of Groningen, Faculty of Theology and Religious Studies, Groningen, The Netherlands

**Keywords:** brain development, cortical subplate, infant, early detection, early intervention, neurodevelopmental disorders, cerebral palsy, low and middle income countries

## Abstract

This paper discusses possibilities for early detection and early intervention in infants with or at increased risk of neurodevelopmental disorders in low- and middle-income countries (LMICs). The brain's high rate of developmental activity in the early years post-term challenges early detection. It also offers opportunities for early intervention and facilitation of school readiness. The paper proposes that in the first year post-term two early detection options are feasible for LMICs: (a) caregiver screening questionnaires that carry little costs but predict neurodevelopmental disorders only moderately well; (b) the Hammersmith Infant Neurological Examination and Standardized Infant NeuroDevelopmental Assessment (SINDA) which are easy tools that predict neurodisability well but require assessment by health professionals. The young brain's neuroplasticity offers great opportunities for early intervention. Ample evidence indicates that families play a critical role in early intervention of infants at increased risk of neurodevelopmental disorders. Other interventional key elements are responsive parenting and stimulation of infant development. The intervention's composition and delivery mode depend on the infant's risk profile. For instance, in infants with moderately increased risk (e.g., preterm infants) lay community health workers may provide major parts of intervention, whereas in children with neurodisability (e.g., cerebral palsy) health professionals play a larger role.

## Introduction

Global mortality in children aged under 5 years decreased by 60% between 1990 and 2020 due to the impact of the United Nations' Millennium Development Goals (1). Unfortunately, this accomplishment was not paralleled by a similar decrease in childhood disability (2). The combination of an increase in surviving children particularly in low- and middle-income countries (LMICs), a rapid population growth in LMICS, and often fragile health care systems in these countries, contributed to a high prevalence of children with neurodevelopmental disabilities (1, 2). It has been estimated that over 53 million children under 5 years had neurodevelopmental disabilities globally in 2016 (3). Over 90% of these children lived in LMICs (1, 4).

The United Nations Convention on the Rights of Persons with Disabilities (2006) and the United Nations Sustainable Development Goal 4 (2015) declared that children with disabilities have the right of inclusive education (5, 6). Nonetheless, UNICEF statistics revealed that many children with disabilities do not receive proper support and adequate education (7). UNICEF's data indicate that children with disabilities are 25% less likely to receive early stimulation and responsive care, 25% less likely to attend early childhood education and 49% more likely to have never attended primary school than children without disabilities (7). In order to improve this situation, it is mandatory that children with neurodevelopmental disorders, such as cerebral palsy (CP), intellectual disability and autism spectrum disorders (ASD), are detected at early age and receive early intervention (2, 8). Early detection and early intervention will result in improved school readiness, as they allow for optimal preparation of family and child so that the child may fully engage in learning experiences at school.

This perspective paper aims to discuss methods available for early detection and early intervention in infants with an increased biological risk of or with a neurodevelopmental disorder (hereafter: infants with R-ND). It pays special attention to those methods that are mostly geared to the health care situation in LMICs. Early detection and early intervention occur in a developmental timeframe that is characterized by abundant brain development. Therefore, the paper first summarizes the developmental changes in the young human brain and its implications for early detection and early intervention. It focuses on the first two postnatal years. The following two sections briefly review knowledge on early detection of and early intervention in infants with R-ND. The last section discusses how early detection and early intervention in infants with R-ND may be achieved best in LMICs. It stresses the importance of family involvement and the need of adaptation to local situations, including cultural habits and beliefs.

## Early human brain development: Opportunities and challenges

### Early human brain development

**Figure 1:**
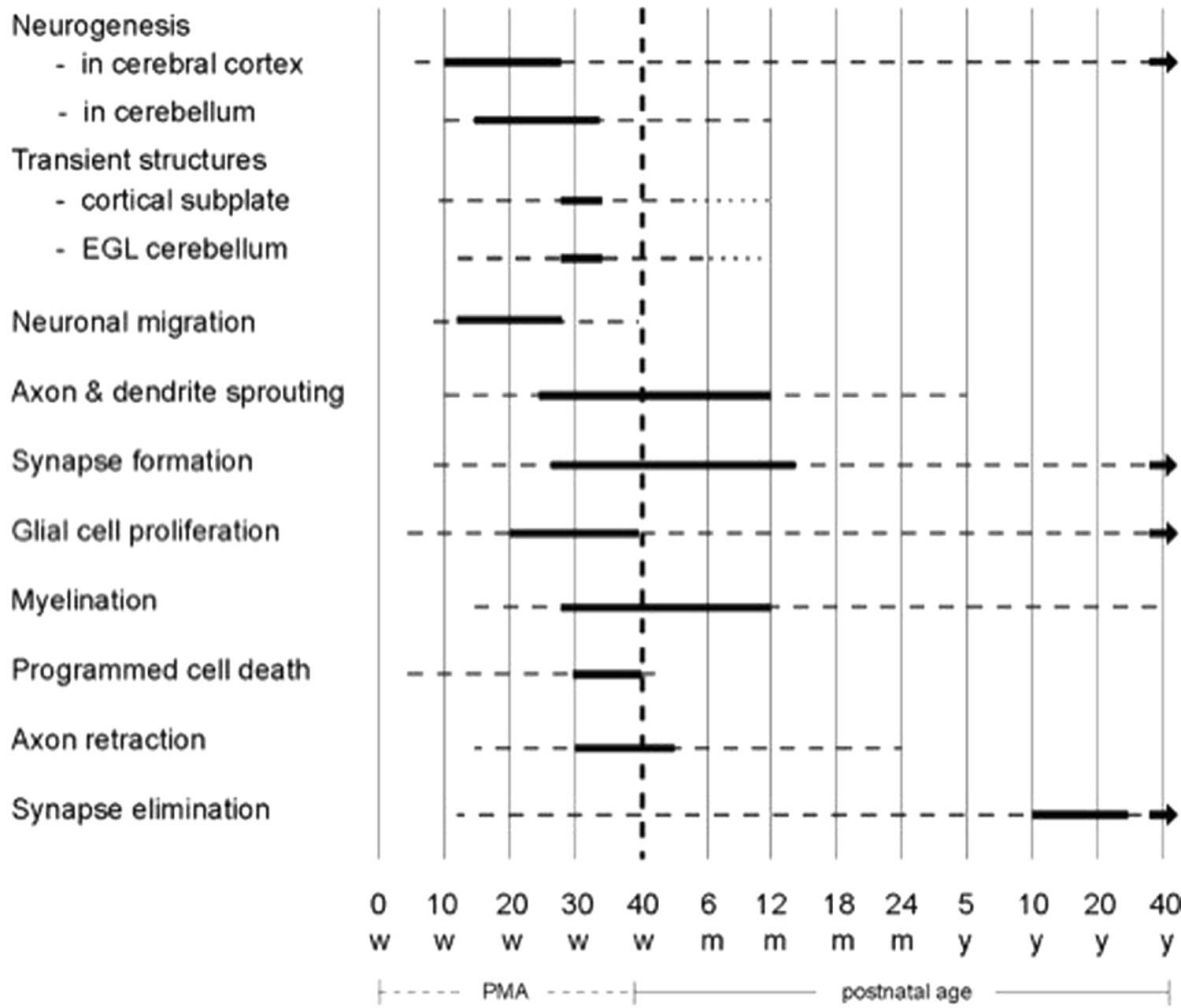
Schematic overview of the developmental processes occurring in the human brain. The bold lines indicate that the processes mentioned on the left side are very active, the broken lines denote that the processes still continue but less abundantly. The diagram is based on reference (9). EGL = external granular layer; m = months; PMA = postmenstrual age; w = weeks; y = years. Figure reproduced with permission from “Early Detection and Early Intervention in Developmental Motor Disorders—from neuroscience to participation” by Mijna Hadders-Algra (ed.) published by Mac Keith Press in its Clinics in Developmental Medicine Series, ISBN number 978-1-911612-43-8 (11).

The development of the human nervous system is a long-lasting and intricate process based on ingenious interactions between genes, environmental information and experience (9). Figure 1 provides an overview of the elementary components of brain development. The majority of neurons and glial cells are generated during prenatal life. Many neurons do not stay at their origin's site but migrate during gestation to their final destination. Neuronal differentiation, synapse production and myelination start early in fetal life to become very active in gestation's last trimester and the first year post-term. Thereafter, these processes continue at a slower pace.

Brain development is not only a matter of production of elements; it also involves massive elimination. About half of generated neurons die through programmed cell death, particularly during gestation's third trimester. Also, axons are initially produced in excess and later partially removed, especially during the end of gestation and the first 3 months post-term. Throughout life, synapses are formed and eliminated, with synapse elimination peaking between the onset of puberty and early adulthood (9).

The combination of production and regression gives rise to temporary structures and connections. Major transient structures are the cortical subplate and the cerebellar external granular layer (EGL; Table 1). The cortical subplate is a temporary structure between the developing white matter and cortical plate. It hosts the first generations of cortical neurons and plays a critical role in cortical development being the major site of neuronal differentiation, synaptogenesis and synaptic activity in the fetal cortex. It receives the first cortical afferents (10). The cortical subplate, which is most prominently present between 28- and 34-weeks postmenstrual age (PMA), mediates fetal behavior. From mid-gestation neurons in the subplate start to die and next generations of migrating cortical neurons begin to populate the cortical plate, i.e., the site of the permanent cortical networks. Around 3 months post-term, the subplate has largely disappeared in the primary motor, sensory and visual cortex, but it takes until the age of 12 months before the subplate has largely dissolved in the frontal, temporal and parietal association areas (9, 10). This means that infant behavior before subplate dissolution is based on activity in the networks in the “fetal” subplate and the cortical plate. First, after the disappearance of major parts of the cortical subplate, infant behavior is mainly mediated by the permanent cortical networks (9, 10). The other significant temporary structure is the cerebellar EGL. The EGL produces the granule cells, the most numerous cells of the brain. The EGL emerges around 15 weeks PMA and is most prominently present between 28- and 34-weeks PMA. Thereafter, it shrinks and disappears completely between 6- and 12-months post-term (9).

**Table 1:** Transient structures in the developing human brain.

Structure	Function	Period of presence
Cortical subplate in primary motor, sensory and visual cortex	- Pivotal role in shaping of permanent circuitries in cortical plate - Mediation of sensorimotor behavior in early life	- Most prominently present at 28–34 week PMA - Largely dissolved around 3 months post-term
Cortical subplate in frontal, temporal and parietal association cortex	- Pivotal role in shaping of permanent circuitries in cortical plate - Mediation of social and motor behavior in early life	- Most prominently present at 28–34 week PMA - Largely dissolved around 12 months post-term
Cerebellar external granular layer	- Production of the granule cells, the most numerous cells of the cerebellum and brain	- Most prominently present at 28–34 week PMA - Dissolving between 6–12 months post-term

For details see references (9) and (10).

As mentioned above, axon development is also characterized by a combination of growth and regression. A well-known example is the axon retraction in the corticospinal tract (11). This tract begins with bilateral projections. Retraction of the ipsilateral projection starts in gestation's last trimester and is largely completed around the age of 2 years (11). This implies that, first at 2 years, the corticospinal tract has achieved its adult configuration with predominantly contralateral projections.

### Implications of early brain development for early detection and early intervention

The brain's developmental activity in the first two years post-term results in specific windows of vulnerability for adverse events, such as inadequate nutrition, preterm birth, or hypoxic-ischemic events (12). The events' unfavorable effect often impacts development in multiple domains, including motor, cognitive, communication and socio-emotional abilities (12). The brain's high developmental activity also has important implications for early detection and early intervention in neurodevelopmental disorders. It offers opportunities and challenges. The brain's great developmental activity generates the opportunity of high neuroplasticity. Neuroplasticity may result in “growing out of dysfunction”. This means that signs of neurological dysfunction that may be present at early age in infants with prenatal, perinatal, or neonatal complications (with or without a brain lesion) may disappear with increasing age (13, 14). Moreover, the high neuroplasticity offers opportunities for early intervention. For instance, it is well known that developmental stimulation in preterm infants results in improved cognitive and motor outcome (15).

The brain's high rate of developmental activity also induces challenges, particularly for early detection of neurodevelopmental disorders. The developmental changes may not only result in resolution of neurological signs, but they may also be associated with the emergence of signs, i.e., “growing into a deficit”. The developing brain usually needs time to express signs of specific neurodevelopmental disorders. The early signs of CP manifest especially from 3 months post-term onwards, i.e., from the time that the cortical subplate in the primary motor and sensory cortex has dissolved (16). Ample evidence has demonstrated that abnormal general movements at 3 months post-term are a powerful predictor of CP (16, 17). The asymmetries of unilateral spastic CP are subtly expressed from 3 to 5 months onwards and become increasingly clear during the rest of the first year when the corticospinal tract reorganizes (18, 19). The early signs of ASD such as impaired social communication, atypical sensory responsivity and repetitive behavior, become clinically predictive from 12 months onwards, i.e., at the age that the cortical subplate has largely disappeared in the cortical association areas and the EGL has vanished (20).

The above described and other early signs of increased risk of disability generally do not allow for the diagnosis of a specific neurodevelopmental disorder. Currently the average age at the diagnosis of CP is 12 months (21), and of ASD, 43 months (22). Nonetheless, it is important to realize that a diagnosis is not needed to start early intervention. Knowing that an infant is at increased risk of neurodevelopmental disorders invokes the need of early intervention (17).

## Early detection of neurodevelopmental disorders

World-wide developmental screening tools are most often used to detect infants with R-ND. Commonly applied methods are caregiver questionnaires [e.g., Parents' Evaluations of Developmental Status (PEDS) (23), Ages and Stages Questionnaire (ASQ) (24)], and the Denver Developmental Screening Test (25). These methods are largely based on attainment of developmental milestones. From the age of 2 years these methods are relatively good in detecting children with developmental delay (26–28). However, their ability to detect children with neurodevelopmental disorders during the first two years is less satisfactory, with sensitivities of 40%–60% and specificities of 59%–77% (29, 30). The most frequently used caregiver questionnaire to detect ASD is the Modified Checklist for Autism in Toddlers [M-CHAT (31)]. In children aged at least 12 months M-CHAT has moderate predictive power in children at increased familial risk of ASD (32).

Five years ago, a systematic review on early prediction of CP indicated that the best methods available for young infants were magnetic resonance imaging (MRI) at term age, and the general movement assessment (GMA) around 3-month post-term (17). In term infants with hypoxic-ischemic encephalopathy, MRI-scans predict CP with sensitivities and specificities of 70%–90% (32). In preterm infants, term-MRI predicts CP with a sensitivity and specificity of 77%–79% (33). GMA is based on the evaluation of the quality of 3 min of general movements in supine. The presence of general movements with seriously reduced movement variation and lacking the age-specific fidgety movements around 3 months post-term predicts CP with a sensitivity and specificity of 91%–98% (16, 34).

The review of Novak et al. (17) also indicated that throughout infancy the Hammersmith Infant Neurological Examination (HINE) is a good instrument to detect CP. It does not only predict CP, but also intellectual disability [Table 2 (39, 40)]. More recently, the Standardized Infant Neurodevelopmental Assessment (SINDA) has been developed. SINDA consists of a neurological, developmental, and socio-emotional scale (36–38). SINDA's neurological scale predicts CP and intellectual disability well; its developmental scale also predicts intellectual disability (Table 2; 15, 32, 45).

**Table 2:** Properties of HINE and SINDA's neurological scale.

**Property**	**HINE**	**SINDA's neurological scale** ^**a**^
Age range (corrected age)	2–3 months - 24 months	6 weeks – 12 months
Domains	- cranial nerve function - posture - movements - muscle tone - reflexes	- spontaneous movement (special attention quality) - cranial nerve function - motor reactions - muscle tone - reflexes
Number of items	26	28
Scoring of items	- ranging from 1 to 4 - criteria for atypical age-dependent	- dichotomous - criteria for atypical not dependent on age
Cut-off for at risk score	varies for different ages and different studies; cut-offs only reported for 3, 6, 9, 12 and 18 months	identical for entire age range: ≤21
Time needed, including administration	<10 min	<10 min
Normative data	not available	present in manual
Reliability	good	good
Prediction of CP		
Sensitivity	- 90%–100%	- 91%–100%
Specificity	- 85%–100%	- 81%–85%
Prediction of CP and/or intellectual disability	intellectual disability	CP and/or intellectual disability
Sensitivity	- 51%–82%	- 83%–89%
Specificity	- 71%–90%	- 94%–96%
Performed by	health professionals	health professionals
Training	*via* website with instructional videos; no manual available	*via* manual and accompanying >160 video clips

^a^ SINDA has two additional scales: a developmental and a socio-emotional scale. The developmental scale has 15 items per months covering cognition, communication, gross and fine motor development. An “at risk” developmental score predicts intellectual disability with a sensitivity of 77% and a specificity of 92%. The socio-emotional scale addresses interaction, emotionality, self-regulation and reactivity. Emotionality and self-regulation predict with sensitivities of 32%–40% and specificities of 85%–98% behavioral and emotional problems at ≥2 years (37).

For details see (35–40).

## Early intervention in infants with or at increased risk of neurodevelopmental disorders

This section focusses on early intervention in infants with R-ND during the first two years. Families play a pivotal role in early intervention (41–43). They form the infants' major environment. Also, family members are the key persons impacting child development through daily interaction during caregiving and play. Details of the intervention approach depend in part on the nature of the infant's risk profile. To this end three groups of infants may be distinguished: (a) infants with prenatal, perinatal, or neonatal complications without a significant brain lesion; (b) infants with a significant brain lesion or neurological signs suggestive of such a lesion; and (c) infants at increased familial risk of ASD.

For the first group of infants, many intervention programs are available (44). Ample evidence exists that sensitive and responsive parent-infant interaction and stimulation of infant development are associated with better family well-being and favorable infant development (11, 32, 45).

Less evidence exists on the effective elements of early intervention in infants with a significant brain lesion (32, 45, 46). Nonetheless, available information suggests that the following key elements are beneficial (32, 45, 46): (a) family involvement; (b) focus on the child's activity domain, i.e., on the child's mobility, learning and knowledge, and communication, and not on impairments such as deviant muscle tone or atypical reflexes; (c) early introduction of assistive devices to promote activities and participation and to prevent contractures and deformities; (d) emphasis on activities and participation of family and child (45). Programs that include these elements are Goals Activity Motor Enrichment (GAME) (47, 48), the Small Step Program (49), COPing with and CAring for infants with special needs (COPCA) (50–52), and - for infants at increased risk of unilateral CP - baby constraint-induced movement therapy (baby-CIMT) (53), and intensive bimanual activities (54). These programs aim to challenge children to explore by self-generated movements with trial and error their own body and the physical and social world.

Knowledge on effective intervention in infants at increased risk of ASD is limited as most intervention studies have been performed in children diagnosed with ASD, implying an age of at least 2.5 years (32). Recent systematic reviews (55–59) suggested but did not prove that in children with ASD, a developmental approach with or without behavioral components is associated with a positive effect on social communication. The evidence on the effect of intervention in infants at increased risk of ASD is very limited (55). The data available suggest that a caregiver-mediated social communication intervention may be associated with improved child attention and social communication and better caregiver responsiveness (55, 60, 61).

## Discussion and conclusion

The rapidly developing brain during infancy imposes challenges for early detection and offers opportunities for early intervention. This is true for high income countries (HICs), but the situation in LMICs is significantly more challenging due to the large number of infants with R-ND in combination with limited resources for early detection and early intervention (62, 63).

Early detection by means of caregiver questionnaires is more cost-effective than that based on testing by professionals. This makes questionnaires (especially PEDS and ASQ) attractive for LMICs despite their less favorable detection properties than assessments by professionals. Nonetheless, barriers such as low caregiver education, illiteracy, and linguistic and cultural diversity may impede general implementation of screening questionnaires (64–67). Assistance by paraprofessional community health workers (CHWs) (68) may reduce these barriers (69) but will increase costs.

The best tools for detection of infants at high risk of neurodevelopmental disorders in the first year post-term are MRI at term, GMA, HINE and SINDA. MRI requires expensive equipment making it less feasible for LMICs. Videorecording of spontaneous movements in GMA is easy and may be performed by caregivers using mobile phones, although educational and linguistic barriers may limit successful recording (70, 71). The latter problem may be solved by videorecording by lay CHWs (68). However, the evaluation of general movement quality requires ample experience, which hampers the implementation of GMA, particularly in LMICs (72, 73). In the future, this situation may change through the application of automated GMA (74–76). Of the best detection tools, HINE and SINDA's neurological scale are the most cost-effective options. HINE and SINDA require the skills of health professionals working in infant health care. Both methods take relatively little time, they do not require an expensive toolkit and they have good predictive properties. HINE covers a larger age range than SINDA. Yet, SINDA's neurological scale has the practical advantages of having a detailed manual and being easier than HINE, as its items and cut-off for “at risk” are independent of infant age (Table 2).

Most early childhood development programs in LMICs focus on health and nutrition in children living in poverty (77). Of course, attention to health and nutrition is quintessential, as health and growth are basic requirements for children to reach their developmental potential. However, the LMIC-literature pays little attention to early intervention in infants at increased risk of neurodevelopmental disorders due to prenatal, perinatal, or neonatal complications, e.g., preterm infants. But it is conceivable that the early intervention strategies that are effective in preterm infants in HICs are also beneficial for preterm infants in LMICs. Actually, the effective strategies to promote development in socially disadvantaged infants in LMICs have large similarities to those applied in preterm infants in HICs (45, 78, 79). Key-elements of both approaches are family involvement, support of caregivers in provision of responsive caregiving, and stimulation of infant development (15, 45, 78, 80). These interventions may be provided by trained lay CHWs to groups of caregivers in the local community with or without home visits by the CHW (81). The home visits may also be replaced by tele-coaching (82). It is conceivable that similar family-community approaches may also work in young children at increased risk of or with ASD. Yet, as described above, evidence on the best intervention approaches in these children is still lacking.

Gradually it is becoming clear which early intervention strategies are beneficial for infants with R-ND due to a significant brain lesion. Essential elements are family involvement, focus on activities and participation of child and family, and prevention of contractures and deformities. Guidance of families with a child with neurodisability is more complex than guidance of families with a preterm infant. It requires more professional effort. Studies performed in LMICs indicate that a combination of caregiver group sessions ran by health professionals in combination with (a) tele-coaching by health professionals and/or (b) home visits by trained lay CHWs may be feasible means to deliver intervention services in infants at increased likelihood of or with neurodevelopmental disorders (82, 83). In the implementation of these early intervention services, it is important to recognize cultural diversity in understanding neurodisability (84). Accordingly, the first steps in early intervention consist of discussing with the family the child's condition, its significance for child, family and community, and the goals of early intervention.

In conclusion, the young brain's neuroplasticity imposes challenges and offers opportunities. It is challenging to detect in the first year infants with R-ND, as the brain needs time to get rid of its temporary structures and to express specific dysfunction. Nonetheless, our hands are not empty: the PEDS, ASQ, HINE and SINDA offer feasible early detection tools for LMICs. Early intervention needs to be geared to the characteristics of child and family. In early intervention for infants with R-ND, the family plays a critical role. In LMICs, families generally are firmly imbedded in the local community, as LMIC-societies function more collectivistic than societies in the individualistic HICs (85). The interdependent societal organization in LMICs may offer specific opportunities for early intervention (84), e.g., through the help of lay CHWs. Cultural integration is a prerequisite for successful early intervention in LMICs (86–88). Adequate early intervention in infants with R-ND will pave the way for school readiness by enhancing attitudes, awareness, knowledge and skills of families and communities, early implementation of assistive devices, and optimizing children's motor, cognitive, communication and socio-emotional skills (1, 8, 45).

## Data Availability

The original contributions presented in the study are included in the article/Supplementary Material, further inquiries can be directed to the corresponding author/s.
